# Low dose nitrite improves reoxygenation following renal ischemia in rats

**DOI:** 10.1038/s41598-017-15058-5

**Published:** 2017-11-03

**Authors:** Kathleen Cantow, Bert Flemming, Mechthild Ladwig-Wiegard, Pontus B. Persson, Erdmann Seeliger

**Affiliations:** 1 Institut für vegetative Physiologie, Center for Cardiovascular Research, Charité – Universitätsmedizin Berlin, Berlin, Germany; 2 Institut für Tierschutz, Tierverhalten und Versuchstierkunde, Freie Universität Berlin, Berlin, Germany

## Abstract

In hypoxic and acidic tissue environments, nitrite is metabolised to nitric oxide, thus, bringing about novel therapeutic options in myocardial infarction, peripheral artery disease, stroke, and hypertension. Following renal ischemia, reperfusion of the kidney remains incomplete and tissue oxygenation is reduced for several minutes to hours. Thus, in renal ischemia-reperfusion injury, providing nitrite may have outstanding therapeutic value. Here we demonstrate nitrite’s distinct potential to rapidly restore tissue oxygenation in the renal cortex and medulla after 45 minutes of complete unilateral kidney ischemia in the rat. Notably, tissue oxygenation was completely restored, while tissue perfusion did not fully reach pre-ischemia levels within 60 minutes of reperfusion. Nitrite was infused intravenously in a dose, which can be translated to the human. Specifically, methaemoglobin did not exceed 3%, which is biologically negligible. Hypotension was not observed. Providing nitrite well before ischemia and maintaining nitrite infusion throughout the reperfusion period prevented the increase in serum creatinine by ischemia reperfusion injury. In conclusion, low-dose nitrite restores renal tissue oxygenation in renal ischemia reperfusion injury and enhances regional kidney post-ischemic perfusion. As nitrite provides nitric oxide predominantly in hypoxic tissues, it may prove a specific measure to reduce renal ischemia reperfusion injury.

## Introduction

In spite of an over thousand year old Chinese text that hints at nitrite as a remedy for angina pectoris, the substance had long been considered to exert few physiological effects, except for causing blue baby syndrome, and being potentially carcinogenic, *via* nitrosamine formation^1,2^. Nutritional sources for nitrite in humans are cured meat products such as hot dogs and – as nitrate is reduced to nitrite by commensal bacteria in the saliva – nitrate-containing vegetables such as beetroots. It is also long known that nitrite is generated by biological decomposition of nitric oxide (NO)^2–5^.

What has become clear in the last decade only, is that *vice versa* nitrite is an important source for NO. Thus, besides the canonical NO generation by NO synthases, NO is formed by reduction of nitrite. This is achieved by a number of proteins including haemoglobin and myoglobin. These proteins’ reductase activity is allosterically regulated by ambient partial pressure of oxygen (pO_2_) and ambient pH in such a way that reductase activity increases in hypoxic and acidic environments. Thus, whereas conventional NO-donors such as nitroglycerin bear a considerable risk for critical hypotension due to generalized vasodilation, nitrite’s mechanism of action provides NO and, thereby, vasodilation predominantly in hypoxic tissues, i.e., “on demand”^3,6,7^.

An ever growing number of studies set out to assess the therapeutic potential of nitrite in a variety of pathophysiological settings. With regard to ischemia-reperfusion injury (IRI) of the heart, liver, and brain, pre-clinical studies have convincingly demonstrated beneficial effects^3,4^. The role of nitrite in *renal* IRI, however, remains to be unraveled. Nitrite dilates renal interlobar arteries under ischemia-mimicking conditions *in vitro*^8^. Two *in vivo* studies reported beneficial effects on read-outs such as plasma creatinine, plasma urea, and histologic scores, yet, a third *in vivo* study did not find any protective effect^9–11^. Remarkably, in these studies, nitrite was never administered during the reperfusion period^9–11^.

Full appraisal of the potentially protective effects of nitrite in renal IRI should include read-outs that mirror pathophysiological key events. Renal tissue hypoperfusion and hypoxia are considered pivotal early elements in the pathophysiology of acute kidney injury of various origins^12–15^. Here, we set out to assess whether nitrite – given in a dose and manner that can be translated into patient treatment – effectively restores renal tissue oxygenation and hemodynamics following renal ischemia.

In accord with safety criteria used for long-term nitrite infusion in humans^7^, an optimum nitrite dose must not induce serious hypotensive episodes and major increases in methaemoglobin (MetHb; the cause of the blue baby syndrome). In addition, the effectiveness of the chosen dosage to enhance hypoxic vasodilation must be ascertained. Our criteria for optimum nitrite administration are as follows: (i) arterial pressure must not drop by more than 15 mmHg under normoxic conditions, (ii) MetHb must not exceed 3%, and (iii) hypoxic vasodilation must be enhanced^7,16^.

## Results

**Figure 1:**
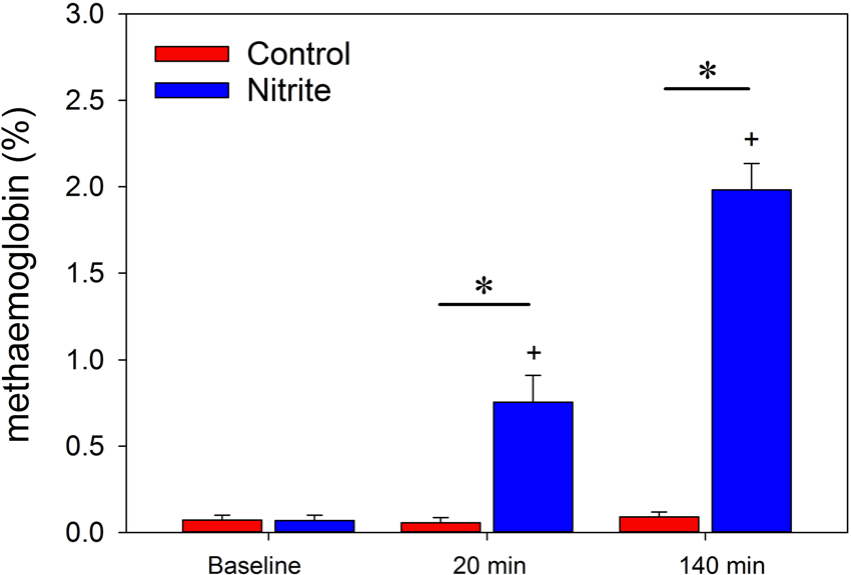
Methaemoglobin at baseline, after 20 min, and after 140 min of continuous nitrite or saline infusion. Data are mean ± SEM, n = 9 for the saline group (control), n = 11 for the nitrite group, *denotes p < 0.05 control vs. nitrite group, + denotes p < 0.05 vs. baseline in the nitrite group. Both groups were exposed to a brief hypoxic challenge and to unilateral ischemia-reperfusion between the blood sampling at 20 min and that at 140 min.

Baseline data on renal hemodynamics and oxygenation before nitrite did not differ between the nitrite group and the saline (volume control) group (Table 1). Also, MetHb did not differ between the groups (Fig. 1). Twenty minutes of continuous nitrite infusion did not change any of the hemodynamic and oxygenation parameters (Table 1); in none of the rats did arterial pressure drop below the set limit of 15 mmHg. MetHb was unchanged in the control group but increased to about 0.8% within 20 minutes of nitrite infusion (Fig. 1). In the nitrite group, MetHb reached about 2.0% after 140 minutes of continuous nitrite infusion, i.e., at the end of the observation period (see Supplemental Fig. 1 for the schedule of the experiments). In none of the rats did MetHb exceed the 3% limit.

**Table 1:** Baseline values and effects of nitrite or saline infusions.

		Baseline		20 min post Saline/Nitrite	
		Saline group	Nitrite group	Saline group	Nitrite group
Arterial blood pressure	mmHg	90.1 ± 3.6	93.8 ± 2.9	90.6 ± 3.7	93.5 ± 3.4
Renal blood flow	mL min^−1^	4.5 ± 0.3	4.7 ± 0.3	4.5 ± 0.2	4.6 ± 0.3
Hindquarter blood flow	mL min^−1^	8.9 ± 1.0	8.9 ± 0.9	8.7 ± 1.0	8.4 ± 0.9
Renal vascular conductance	mL min^−1^ mmHg^−1^	0.051 ± 0.004	0.051 ± 0.004	0.050 ± 0.003	0.049 ± 0.004
Hindquarter vascular cond.	mL min^−1^ mmHg^−1^	0.101 ± 0.013	0.094 ± 0.013	0.098 ± 0.012	0.093 ± 0.012
Cortical tissue pO_2_	mmHg	21.4 ± 3.6	23.9 ± 3.4	22.5 ± 3.5	22.7 ± 3.3
Medullary tissue pO_2_	mmHg	16.4 ± 3.2	17.6 ± 2.7	17.4 ± 2.9	17.4 ± 3.7
Cortical laser flux	% change			2.0 ± 3.3	−3.3 ± 3.5
Medullary laser flux	% change			1.3 ± 5.8	3.0 ± 6.4

Data are mean ± SEM, n = 9 for the saline group (control), n = 11 for the nitrite group. There were no significant differences between the groups at baseline and after 20 min of nitrite or saline infusion; neither nitrite nor saline infusion resulted in significant changes vs. the respective baseline values. Please note that data on cortical and medullary laser flux are given as percentage change from baseline only, because laser fluxmetry does not provide absolute data on blood perfusion.

**Figure 2:**
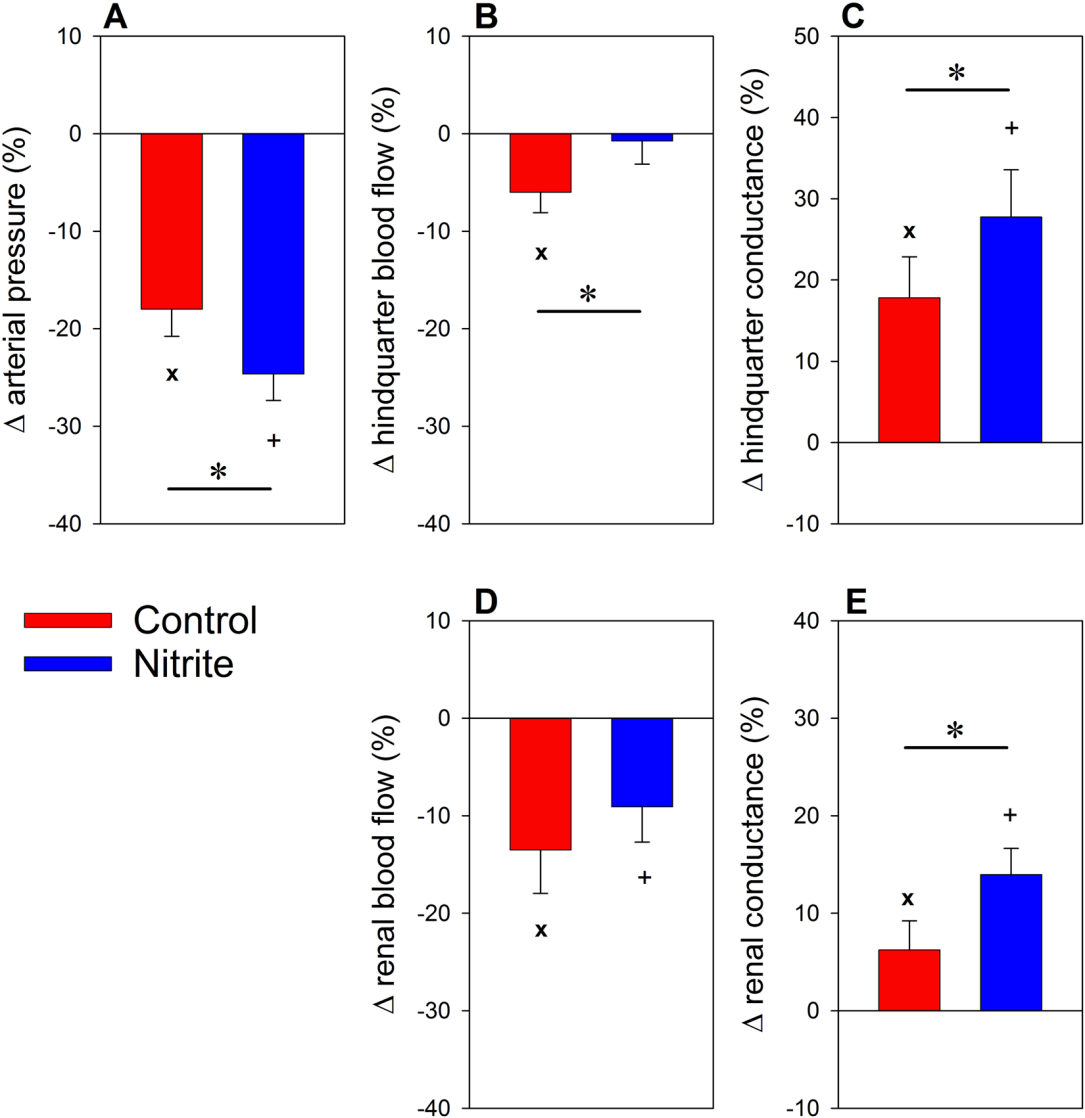
Response of arterial pressure (panel A), hindquarter blood flow (**B**), hindquarter vascular conductance (**C**), total renal blood flow (**D**), and renal vascular conductance (**E**) to a brief hypoxic challenge (inspiratory fraction of oxygen 10%). Data are percentage changes at the end of 100 s of hypoxia as related to the values recorded immediately before commencement of the hypoxia intervention (mean ± SEM, n = 9 for the saline group [control], n = 11 for the nitrite group); *denotes p < 0.05 control vs. nitrite group, **x** denotes p < 0.05 normoxia vs. hypoxia in the control group, +denotes p < 0.05 normoxia vs. hypoxia in the nitrite group.

The effectiveness of the nitrite infusion to enhance hypoxic vasodilation was tested by briefly lowering the inspiratory fraction of oxygen (FiO_2_) from normoxia (21%) to 10%. In the control group, 100 seconds of hypoxia decreased arterial blood pressure by hypoxic vasodilation, which was pronounced in the hindquarter as compared to the renal circulation (Fig. 2). Nitrite significantly enhanced the hypotensive effect by augmenting hypoxic vasodilation in both the hindquarter and kidneys.

**Figure 3:**
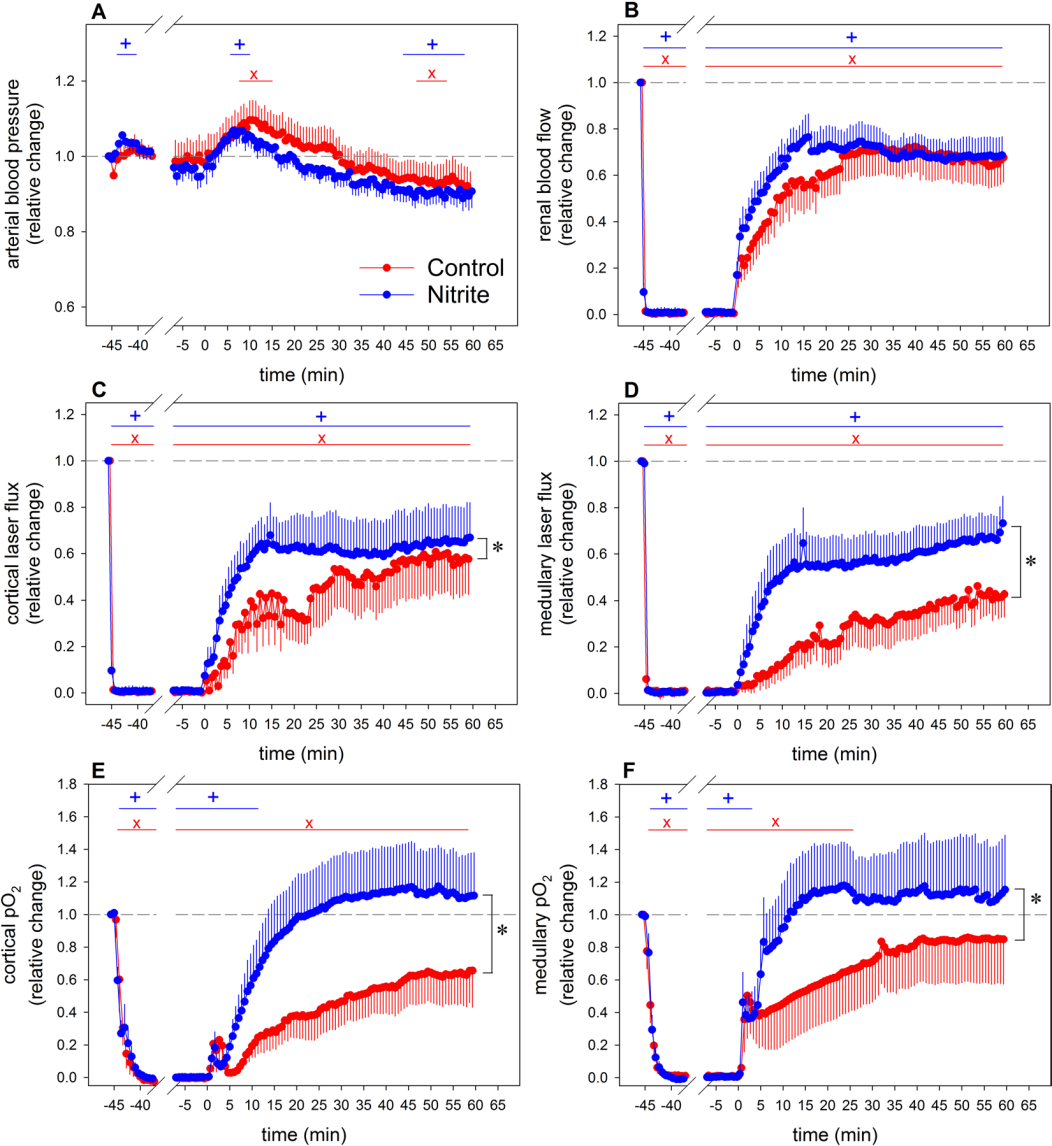
Response of arterial blood pressure (panel A), total renal blood flow (**B**), cortical laser flux (**C**), medullary laser flux (**D**), cortical tissue pO_2_ (**E**), and medullary tissue pO_2_ (**F**) to 45 min of unilateral warm renal ischemia (from time −45 min to time 0 min) followed by 60 min of reperfusion. Data are relative changes as related to the values recorded immediately before commencement of the renal ischemia (mean ± SEM; n = 9 for the saline group [control], n = 11 for the nitrite group); *denotes p < 0.05 control vs. nitrite group, significance bars indicate data that significantly differ from their pre-ischemia values, with **x** denoting p < 0.05 in the control group and +denoting p < 0.05 in the nitrite group.

The effects of IRI on renal hemodynamics and tissue oxygenation are depicted by Fig. 3. In the control group, total renal blood flow as well as local cortical and medullary perfusion that were nil during the occlusion of the renal artery and vein, started to increase upon unclamping. Restoration was sluggish and incomplete, even after 60 min of reperfusion. Cortical and medullary pO_2_ that also approached zero during the occlusion, showed an immediate increase upon its cessation, reaching a small transient peak. Thereafter, cortical pO_2_ increased slowly and did not reach pre-occlusion values. Conversely, medullary pO_2_ took on values indistinguishable from control levels already 25 min after reperfusion. Unilateral IRI increased serum creatinine significantly by 12.0 ± 4.6 micromol/L from 53.9 ± 2.9 micromol/L at baseline to 65.9 ± 6.2 micromol/L at the end of the observation period.

Overall, continuous nitrite infusion improved post-ischemic perfusion and oxygenation, but distinct differences were seen between total renal and local perfusion and, most remarkably, between perfusion and tissue pO_2_ (Fig. 3). Local reperfusion in the cortex and, even more so, in the medulla were significantly augmented by nitrite, while its effect on total renal blood flow did not reach statistical significance. Nitrite rapidly restored tissue pO_2_ in both the cortex and the medulla to pre-ischemic levels. Serum creatinine did not change significantly (3.3 ± 4.7 micromol/L) from baseline (54.3 ± 2.4 micromol/L) to the end of the observation period (57.6 ± 5.8 micromol/L).

## Discussion

This pre-clinical study shows that nitrite – given in a dose and manner that can be translated into patient treatment – effectively restores renal tissue oxygenation following renal ischemia. The improved re-oxygenation most probably relies on the nitrite-reductase pathway for NO generation. It provides NO and, thereby, vasodilation “on demand”, yet other renal effects of NO including an improved supply-demand ratio for oxygen appear to play an additional role in the reno-protective effect of continuous low-dose nitrite administration.

In line with pathophysiologic key events in acute kidney injuries of various other origins, renal tissue hypoperfusion and hypoxia during early reperfusion play a prominent role in the pathophysiology of renal IRI^12–15,17–22^. Tissue hypoxia following ischemia relies, at least in part, on imbalance between vasodilatory factors such as NO, and vasoconstrictive factors, e.g., reactive oxygen species and 20-hydroxyeicosatetraenoic acid, among many others^12,13,15,17^.

We observed that, in the control group, renal tissue perfusion increased slowly upon cessation of the ischemia and did not reach pre-occlusion values within 60 min of reperfusion (Fig. 3). Both cortical and medullary tissue pO_2_ showed a small transient peak upon unclamping, in line with a previous study^17^. This most probably mirrors an increase in the supply-demand ratio during a short period where reperfusion supplies oxygen, while oxygen consuming tubular reabsorption has not yet started again. Thereafter, cortical pO_2_ increased slowly and did not reach pre-occlusion values, whereas medullary pO_2_ was restored within about 25 min of reperfusion. This points at differences in post-ischemic oxygen supply-demand ratio, probably related to lower overall osmolyte reabsorption of medullary than cortical tubules.

Restoring post-ischemic oxygenation is an obvious target for alleviating IRI, and, given that imbalance between vasoconstrictive factors and vasodilatory NO plays a key role, providing NO “on demand” can be considered a rewarding approach. Nitrite infusion, indeed, increased post-ischemia tissue perfusion and oxygenation (Fig. 3). Nitrite significantly augmented local cortical reperfusion and, even more so, medullary reperfusion, while its effect on total renal blood flow did not reach statistical significance. This indicates redistribution of intrarenal perfusion towards the medulla, and a possible pooling within the microcirculation^12,22^. The latter enhances laser-Doppler probes signals, as this technique assesses erythrocytes’ velocity and the density of erythrocytes per tissue volume^23^. Nitrite rapidly restored tissue pO_2_ to pre-ischemic levels. The discrepancy between perfusion and tissue pO_2_ indicates an altered oxygen supply-demand ratio. NO is known to decrease tubular sodium reabsorption and, thereby, oxygen consumption^24^. In addition, NO enhances sodium reabsorption efficiency^25^.

The rapid and full restoration of tissue oxygenation in the nitrite group parallels the finding that the classical marker for renal function, serum creatinine, did not increase significantly. Conversely, renal IRI increased serum creatinine in the control group by about 12 micromol/L. As we exposed only the left kidney to IRI, creatinine increase is less than for rats subjected to bilateral kidney IRI or combined unilateral IRI with contralateral nephrectomy^9,11,17^, in line with previous studies^26,27^.

Former studies on the efficacy of nitrite in alleviating renal IRI applied nitrite (or nitrate) in a manner or dosing that cannot be transferred to the patient. For instance, nitrite was applied topically on the kidney surface, or in high doses. The present protocol aimed at only moderately increased nitrite levels, in accord with safety criteria used for long-term nitrite infusion in humans^7^. Continuous i.v. infusion (that includes the reperfusion period) is preferred over bolus injections, intraperitoneal, or topical administration on the kidney surface^9–11^, to allow nitrite distribution among fluid compartments and to take into account complex kinetics of interactions among nitrite, haemoglobin, nitrate, and NO in humans^28^. In patients, serious hypotensive episodes must be precluded and the increase in MetHb must be limited. Our dosage for nitrite infusion met the set criteria. First, nitrite did not result in a significant decrease in arterial blood pressure under normoxia (Table 1), and in none of the rats did arterial pressure drop by 15 mmHg or more. Second, MetHb increased by about 2.0% within the observation period of 140 minutes (Fig. 1), and in none of the rats did MetHb exceed the 3% limit. Such minute MetHb levels are deemed biologically negligible^7^.

Third, the effectiveness of the chosen dosage to enhance hypoxic vasodilation was ascertained. In the control group, hypoxia decreased arterial pressure by hypoxic vasodilation, which, in accord with previous results^16,23^, is pronounced in non-renal vascular beds (exemplified here by the hindquarter) as compared to the renal vasculature (Fig. 2). Nitrite significantly enhanced the hypotensive effect by augmenting hypoxic vasodilation in both the hindquarter and the kidneys. This result is in line with studies in human beings, in whom low-dose nitrite infusions increased forearm blood flow during hypoxia, but not during normoxia^29^. Indeed, the presented data confirm that the nitrite-reductase pathway provides NO and, thereby, vasodilation “on demand”^3,6,7,16^.

Safety and feasibility of intravenous sodium nitrite infusion in healthy humans is already proven^7^. Here we show that nitrite, given to rats in an according manner, markedly alleviates renal IRI, with regard to local tissue oxygenation, regional perfusion and serum creatinine. Thus, these findings underscore the beneficial effects of low-dose nitrite infusion on renal oxygenation in a model of contrast-induced acute kidney injury^16^. Low-dose nitrite administration was recently also shown to prevent lethality in a model of crush-syndrome^30^. Our findings add to the studies in patients suffering from a variety of cardiovascular disorders, which are currently under way. Patients with endothelial dysfunction (e.g. diabetics) may profit most^2,3^, but attention must be paid in individuals prone to generalised hypoxia, as serious hypotension may occur. According to recent studies in human beings, dietary intakes of nitrate and nitrite lower the risk of hypertension and chronic kidney disease, and patients suffering from chronic kidney diseases profit from increased dietary nitrite intake^31,32^. Yet, in spite of the encouraging findings, it appears too early to generally recommend nitrite administration – or consuming hot dogs or beetroot juice – when at threat for renal IRI.

## Materials and Methods

Investigations were performed on 20 male Wistar rats (4–5 month of age; average body mass [BM] 400 g; Harlan-Winkelmann, Borchen, Germany). The studies were approved by Berlin’s State Office of Health and Social Affairs in accordance with the German Animal Protection Law and the experiments were carried out in accordance with the approved guidelines. The rats were housed under standard conditions with environmental enrichment and were allowed food and water intake *ad libitum* until administration of the anesthetic. For anesthesia, urethane solution (Sigma-Aldrich, Steinheim, Germany; 20% in distilled water) was intraperitoneally injected at 6 mL/kg of BM. This approach provides anesthesia throughout the surgical preparation and the examination and leaves cardiovascular and respiratory reflexes largely undisturbed. The rats were positioned on a heating table to maintain their body temperature at 37 °C.

### Surgical Preparation

A tracheal cannula was inserted to facilitate spontaneous breathing. A femoral artery catheter was used to monitor arterial blood pressure via a transducer (DTXX; Viggo-Spectramed, Swindon, UK) connected to an amplifier (Gould, Valley View, OH). This catheter also served for continuous saline infusion (1 mL/h) and for intermittent blood sampling. The abdomen was opened through a midventral incision; during surgery and examination, the abdominal cavity was filled with isotonic saline (37 °C). Two ultrasound transit time difference flow probes (1RB; Transonic Systems, Ithaca, NY) were positioned by means of micromanipulators, one around the left renal artery, the other around the infrarenal aorta, for absolute measurements (in mL/min) of total renal blood flow and hindquarter blood flow, respectively. Two combined optical laser-Doppler flux and pO_2_ probes (OxyFlo/OxyLite; Oxford Optronics, Oxford, UK) were inserted by means of micromanipulators into the renal cortex and the medulla (probe tips about 1.5 mm and 3–4 mm below the capsule, respectively) to obtain absolute values of tissue pO_2_ (in mmHg) and relative changes in tissue perfusion (laser flux). A soft suture sling armed with an elastic tube around the sling’s legs was loosely placed around the left renal artery and vein for later induction of ischemia. Finally, a jugular vein catheter was inserted that was used for infusions.

### Experimental Procedures

As depicted by Supplemental Fig. 1, after completion of the surgical preparation, baseline values of hemodynamic and oxygenation parameters and a blood sample were obtained during infusion of saline (0.67 mL/h per kg BM via the jugular vein catheter). MetHb was measured by ABL 520 apparatus (Radiometer, Copenhagen, Denmark) and serum creatinine by Creatinine Analyzer II (Beckman Instruments, Galway, Ireland). The withdrawn blood volume was replaced by an equal amount of balanced electrolyte solution (E153; Serumwerk, Bernburg, Germany).

Thereafter, in one group of rats (n = 11), the infusion was switched to nitrite, whereas in the other group (n = 9), the saline infusion was continued (control). While the infusion rate was maintained at 0.67 mL/h per kg BM, the nitrite dose for the initial 10 minutes was 0.172 mg/h per kg BM (solution of 225 mmol/L of sodium nitrite [Roth GmbH, Karlsruhe, Germany] in distilled water), which was followed by a nitrite dose of 0.057 mg/h per kg (solution of 75 mmol/L of sodium nitrite in 0.45% NaCl) that was continued until the end of the experiment^16^.

Twenty minutes after initiating the infusion of nitrite (or continuation of saline, respectively), a second blood sample was taken. Subsequently, a brief period of hypoxia was induced to assess the effect of nitrite on hypoxic vasodilation by reducing FiO_2_ from 21% (room air) to 10% (hypoxia); the FiO_2_ was monitored by means of a gas analyzer (ML206; ADInstruments, Dunedin, New Zealand). Hundred seconds after initiating hypoxia, the FiO_2_ was restored to 21%, which was followed by a recovery period of 300 seconds.

Thereafter, ischemia of the left kidney was initiated by drawing the legs of the suture sling and fixing them above the elastic tube by means of a lightweight bulldog clamp. Forty-five minutes later, the sling was removed to allow reperfusion. The completeness of the occlusion and re-opening of the renal artery and vein was visually controlled. Sixty minutes after initiating the reperfusion, a last blood sample was taken.

### Calculations and Statistical Analyses

To distinguish changes in blood flow brought about by changes of arterial pressure *via* passive vessel distension from that brought about by vasomotor actions, conductance values (the inverse of resistance) were calculated by dividing flow data by the present arterial pressure: renal vascular conductance = renal blood flow/arterial pressure, and hindquarter conductance = hindquarter blood flow/arterial pressure. Relative values and percentage changes were obtained by relating the absolute data during/after an intervention to the absolute data obtained immediately before the respective intervention.

Data are given as mean ± SEM. Statistical analyses were done using the Student *t* test and general linear model analysis of variance (GLM-ANOVA) followed by Dunnett’s multiple comparison procedure, respectively, with a significance level of P < 0.05 using Number Cruncher Statistical Software (Hintze, Kaysville, UT).

### Data availability

All data generated or analysed during this study are included in this published article.

## Electronic supplementary material

supplemental figure 1

## References

[1] Khatri J, Mills CE, Maskell P, Odongerel C, Webb AJ (2017). It is Rocket Science - Why dietary nitrate is hard to beet! Part I: Twists and turns in the realisation of the nitrate-nitrite-NO pathway. Br J Clin Pharmacol.

[2] Parthasarathy DK, Bryan NS (2012). Sodium nitrite: the “cure” for nitric oxide insufficiency. Meat Sci.

[3] Omar SA, Webb AJ, Lundberg JO, Weitzberg E (2016). Therapeutic effects of inorganic nitrate and nitrite in cardiovascular and metabolic diseases. J Intern Med.

[4] Mills CE, Khatri J, Maskell P, Odongerel C, Webb AJ (2017). It is rocket science - why dietary nitrate is hard to Beet!part II: further mechanisms and therapeutic potential of the nitrate-nitrite-NO pathway. Br J Clin Pharmacol.

[5] Gladwin MT (2005). The emerging biology of the nitrite anion. Nat Chem Biol.

[6] Gladwin MT, Grubina R, Doyle MP (2009). The new chemical biology of nitrite reactions with hemoglobin: R-state catalysis, oxidative denitrosylation, and nitrite reductase/anhydrase. Acc Chem Res.

[7] Pluta RM (2011). Safety and feasibility of long-term intravenous sodium nitrite infusion in healthy volunteers. PLoS One.

[8] Liu M (2015). Nitrite-mediated renal vasodilatation is increased during ischemic conditions via cGMP-independent signaling. Free Radic Biol Med.

[9] Basireddy M, Isbell TS, Teng X, Patel RP, Agarwal A (2006). Effects of sodium nitrite on ischemia-reperfusion injury in the rat kidney. Am J Physiol Renal Physiol.

[10] Milsom AB (2010). Role for endothelial nitric oxide synthase in nitrite-induced protection against renal ischemia-reperfusion injury in mice. Nitric Oxide.

[11] Tripatara P (2007). Nitrite-derived nitric oxide protects the rat kidney against ischemia/reperfusion injury *in vivo*: role for xanthine oxidoreductase. J Am Soc Nephrol.

[12] Evans RG (2013). Haemodynamic influences on kidney oxygenation: clinical implications of integrative physiology. Clin Exp Pharmacol Physiol.

[13] Heyman SN, Evans RG, Rosen S, Rosenberger C (2012). Cellular adaptive changes in AKI: mitigating renal hypoxic injury. Nephrol Dial Transplant.

[14] Fähling M, Seeliger E, Patzak A, Persson PB (2017). Understanding and preventing contrast-induced acute kidney injury. Nat Rev Nephrol.

[15] Zuk A, Bonventre JV (2016). Acute Kidney Injury. Annu Rev Med.

[16] Seeliger E (2014). Low-dose nitrite alleviates early effects of an X-ray contrast medium on renal hemodynamics and oxygenation in rats. Invest Radiol.

[17] Hoff U (2011). Inhibition of 20-HETE synthesis and action protects the kidney from ischemia/reperfusion injury. Kidney Int.

[18] Peer V, Abu HR, Berman S, Efrati S (2016). Renoprotective effects of DNAse-I treatment in a rat model of ischemia/reperfusion-induced acute kidney injury. Am J Nephrol.

[19] Pohlmann A (2013). High temporal resolution parametric MRI monitoring of the initial ischemia/reperfusion phase in experimental acute kidney injury. PLoS One.

[20] Abdelkader A (2014). Renal oxygenation in acute renal ischemia-reperfusion injury. Am J Physiol Renal Physiol.

[21] Ergin B, Heger M, Kandil A, Demirci-Tansel C, Ince C (2017). Mycophenolate mofetil improves renal hemodynamics, microvascular oxygenation, and inflammation in a rat model of supra-renal aortic clamping-mediated renal ischemia reperfusion injury. Clin Exp Pharmacol Physiol.

[22] Snoeijs MG (2010). Acute ischemic injury to the renal microvasculature in human kidney transplantation. Am J Physiol Renal Physiol.

[23] Grosenick D (2015). Detailing renal hemodynamics and oxygenation in rats by a combined near-infrared spectroscopy and invasive probe approach. Biomed Opt Express.

[24] Garvin JL, Herrera M, Ortiz PA (2011). Regulation of renal NaCl transport by nitric oxide, endothelin, and ATP: clinical implications. Annu Rev Physiol.

[25] Laycock SK (1998). Role of nitric oxide in the control of renal oxygen consumption and the regulation of chemical work in the kidney. Circ Res.

[26] Mitaka C (2014). Effects of atrial natriuretic peptide on inter-organ crosstalk among the kidney, lung, and heart in a rat model of renal ischemia-reperfusion injury. Intensive Care Med Exp.

[27] Tulafu M (2014). Atrial natriuretic peptide attenuates kidney-lung crosstalk in kidney injury. J Surg Res.

[28] Hon YY, Sun H, Dejam A, Gladwin MT (2010). Characterization of erythrocytic uptake and release and disposition pathways of nitrite, nitrate, methemoglobin, and iron-nitrosyl hemoglobin in the human circulation. Drug Metab Dispos.

[29] Maher AR (2008). Hypoxic modulation of exogenous nitrite-induced vasodilation in humans. Circulation.

[30] Murata I (2017). Low-dose sodium nitrite fluid resuscitation prevents lethality from crush syndrome by improving nitric oxide consumption and preventing myoglobin cytotoxicity in kidney in a rat model. Shock.

[31] Bahadoran Z (2016). Association between dietary intakes of nitrate and nitrite and the risk of hypertension and chronic kidney disease: Tehran lipid and glucose study. Nutrients.

[32] Kemmner S (2017). Dietary nitrate load lowers blood pressure and renal resistive index in patients with chronic kidney disease: A pilot study. Nitric Oxide.

