# Publisher Correction: Low dose nitrite improves reoxygenation following renal ischemia in rats

**DOI:** 10.1038/s41598-018-19651-0

**Published:** 2018-01-24

**Authors:** Kathleen Cantow, Bert Flemming, Mechthild Ladwig-Wiegard, Pontus B. Persson, Erdmann Seeliger

**Affiliations:** 1 Institut für vegetative Physiologie, Center for Cardiovascular Research, Charité – Universitätsmedizin Berlin, Berlin, Germany; 2 Institut für Tierschutz, Tierverhalten und Versuchstierkunde, Freie Universität Berlin, Berlin, Germany

Correction to: *Scientific Reports* 10.1038/s41598-017-15058-5, published online 03 November 2017

The original HTML version of this Article contained a typographical error in the publication date ‘03 November 2017’ which was incorrectly given as ‘06 November 2017’. This has now been corrected in the HTML version of the Article.

