# Supplementary material for: Low dose nitrite improves reoxygenation following renal ischemia in rats
Source: Sci Rep. 2017 Nov 3;7:14597. doi: 10.1038/s41598-017-15058-5 (PMC5668317; doi:10.1038/s41598-017-15058-5)
Supplement: Supplementary file 1 — supplemental figure 1 [file 41598_2017_15058_MOESM1_ESM.pdf]

Low dose nitrite improves reoxygenation following renal ischemia in rats

Kathleen Cantow, Bert Flemming, Mechthild Ladwig-Wiegard, Pontus B. Persson, and Erdmann Seeliger

Supplemental Figure 1

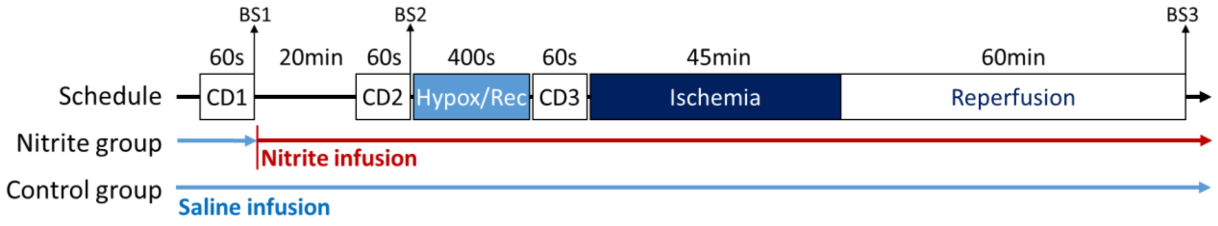

Scheme depicting the schedule of the experiments (please note: times are not to scale). After completion of the surgical preparation, registration of control data and blood sampling (CD1 and BS1) took place to obtain baseline data. Thereafter, nitrite infusion was initiated in the nitrite group while saline infusion was continued in the control group. Twenty minutes later, another set of control data and blood sample was obtained (CD2 and BS2), before a brief hypoxic challenge and recovery (Hypox/Rec; inspiratory O_2 fraction of 10% for 100 sec, followed by 300 sec recovery at inspiratory O_2 fraction of 21%) was performed. Following another registration of control data (CD3), ischemia of the left kidney was initiated and maintained for 45 min, which was followed by 60 min of reperfusion, at the end of which a last blood sample (BS3) was taken.
